# Exploring adolescent mental health during the COVID-19 crisis – strengths and difficulties

**DOI:** 10.3389/fpubh.2024.1357766

**Published:** 2024-04-04

**Authors:** Johanna K. Loy, Janina Klam, Jörg Dötsch, Julia Frank, Stephan Bender

**Affiliations:** ^1^Department of Child and Adolescent Psychiatry, Psychosomatics and Psychotherapy, Faculty of Medicine and University Hospital Cologne, University of Cologne, Cologne, Germany; ^2^Department of Pediatric and Adolescent Medicine, Faculty of Medicine and University Hospital Cologne, University of Cologne, Cologne, Germany; ^3^Institute of Medical Statistics and Computational Biology, Faculty of Medicine, University of Cologne, Cologne, Germany

**Keywords:** COVID-19 pandemic, adolescents, mental health, SDQ-questionnaire, crisis, mental health assessment

## Abstract

**Introduction:**

The SARS-CoV-2 pandemic has significantly impacted children and adolescents, leading to mental health challenges. Knowledge on their resources and difficulties is crucial and there is a need for valid instruments to assess their psychosocial condition especially in this exceptional situation. We assessed psychopathological symptoms using the SDQ during the pandemic, comparing to pre-pandemic data. Our study aims to understand adolescents’ strengths and difficulties amidst COVID-19, evaluating the SDQ’s utility in crisis settings.

**Methods:**

Within the German school-based surveillance study (“B-Fast”), we assessed behavioral strengths and difficulties in 664 adolescents aged 11–17 years during the peak of the German COVID-19 pandemic using the validated Strengths and Difficulties Questionnaire (SDQ) for both external and self-assessed data collection. Data were collected between November 2020 and April 2021. We compared self-assessed SDQ-scores to pre-pandemic data from a comparable sample and examined adolescent classification as “normal” or “borderline/abnormal” based on both external and self-assessed SDQ subscale scores using established cut-off values. Additionally, we conducted sex and rater-based score comparisons.

**Results:**

In our study, we observed a significant worsening of “Emotional Symptoms” compared to pre-pandemic levels, while “Conduct Problems” and “Prosocial Behavior” showed improvement. Variations in classification to “normal” and “abnormal” emerged when applying German versus British cut-off values. Females scored higher on “Emotional Symptoms” while males scored higher on “Hyperactivity Symptoms.” Correlations between external and self-assessed SDQ ratings ranged from 0.43 (*p* < 0.001) for “Prosocial Behavior” among girls to 0.62 (*p* < 0.001) for “Peer Problems” among boys, indicating moderate to high consistency.

**Discussion/conclusion:**

Our study contributes to understanding the psychosocial impact of the COVID-19 pandemic on German adolescents. Compared to other symptoms, we observed a particular worsening in “Emotional Symptoms” based on our data. Despite the moderate correlation between parental and self-reported evaluations, there appears to be a certain discrepancy in the perception of adolescent quality of life. Therefore, it seems prudent to assess both the external and self-reported evaluations and amalgamate the results from both parties to obtain a comprehensive problem profile of the individual. These findings underscore the importance of using country-specific cutoff values and reaffirm the utility of the SDQ as a valuable assessment tool, even within the unique circumstances posed by a pandemic.

## Introduction

Since its outbreak and global spread, the SARS-CoV-2-pandemic has been a major challenge to the world’s population and a major burden to health, social and economic systems worldwide, especially threatening vulnerable groups, such as children and adolescents (1). While it is currently believed that COVID-19 infections among them mostly result in mild courses with few complications (which is not yet conclusively understood and currently still under investigation), numerous studies have demonstrated the devastating indirect effects of this pandemic on the well-being of children and adolescents. (2–14). For example, In the nationwide representative COPSY study, the mental health and quality of life of children and adolescents during the pandemic were examined and compared with pre-pandemic data. Results indicated a high level of distress, diminished quality of life, and a significant increase in mental health issues (11–13). These findings align with a systematic review conducted by Loades and colleagues (7), which explored the repercussions of social isolation on children’s mental health during the COVID-19 pandemic in several countries. The review revealed elevated levels of depression and anxiety persisting even beyond the period of isolation. Similarly, Fong and Iarocci (5) emphasized the substantial negative impact of COVID-19 and related social isolation/quarantining practices on child anxiety, post-traumatic stress disorders and fear symptoms. Potential reasons for these severe impacts of the pandemic on children, adolescents and their families include the drastic mitigation measures that severely limit social and educational opportunities for personal development, constant fear for the own health and that of parents and loved ones, potentially threatening economic consequences and the loss of a predetermined daily structure (15–17).

A Lancet article by Jiao et al. (18) delved into the implications of extended home confinement and school closures during the COVID-19 pandemic on children’s mental health. It highlights concerns about increased anxiety, depression, and psychological distress among children due to disrupted routines and limited social interactions. Drawing on evidence from previous outbreaks, the study emphasized the urgent need for targeted interventions to address mental health concerns during public health emergencies. Additionally, it underscored the critical role of parents, schools, and communities in supporting children’s mental well-being during crises.

It has been shown that during COVID-19 pandemic a significant increase of psychological distress, Covid-19 anxiety syndrome, loneliness, fatigue, and worry has been observed across different populations (19–22). Overall however, several studies so far during the SARS-CoV-2-pandemic in children and adolescents focused on physical health or measures of quality of life (11) but still less on psychopathological symptoms. To fill this gap, we conducted a dimensional approach to assess the frequency and number of psychopathological symptoms with the SDQ-questionnaire, which also delves into aspects of psychopathology and offers self-assessed as well as external ratings. This approach was deemed important, as the WHO reports increased rates of psychiatric disorders (23). Good agreement between an SDQ assessment and the respective clinical diagnoses has already been demonstrated, in principle making the SDQ a useful screening tool for emotional and behavioral strengths and difficulties in children and adolescents (24). We wanted to find out whether the SDQ, instead of the lengthy Child Behavior Checklist (CBCL) questionnaire, is also suitable for use during a pandemic, assessing to what extent it can effectively serve as a time-efficient and cost-effective tool to serve as a proxy for potential psychopathological symptoms. Furthermore, differentiated knowledge in terms of a profile of psychopathological aspects is needed. This allows an assessment of how various domains of psychopathology were affected by the pandemic stress. The COSMO study (12) has reported important data about increased emotional problems in children and adolescents during the SARS-Cov-2 pandemics using the SDQ, however, no data on other psychopathological dimensions or consequences for how many subjects are detected as “abnormal” were reported in this study.

More specifically, the study at hand aims to examine how suitable the SDQ is to measure psychopathological symptoms among children and adolescents aged 11–17 years during the SARS-CoV-2 pandemic. Further, with the data of a convenience sample, the study aims to convey an impression on children’s and adolescents’ strengths and difficulties during the pandemic. To do so we are (1) providing a description of their SDQ-scores, highlighting the strengths and difficulties faced by the adolescents in the cohort. In addition, we are (2) using the externally- and self-assessed ratings for comparative analyses. This includes the comparison of (a) these SDQ-scores with normative pre-pandemic data, (b) the application of different country-specific cut-off-values to the results for a classification to normal and borderline/abnormal, (c) outcomes of male and female participants across the different SDQ subscales of the Questionnaire separately for external and self-ratings and (d) results of the external and self-assessed SDQ ratings separately for males and females. All results are interpreted and contextualized against the backdrop of previous knowledge about the psychological situation of adolescents in order to assess the validity of the SDQ during global crises.

## Methods

The National Research Network of the university hospitals in Germany initiated the B-Fast Project with the goal to collect and bundle pandemic information and knowledge to contribute to the management and control of the SARS-CoV-2 pandemic, especially in institutions of children’s everyday life. The project received funding from the Federal Ministry of Education and Research. The study was approved by the ethics committee of the medical faculty of the University of Cologne and the respective ethics committees of all participating study sites and is registered with the German Clinical Trials Register (http://www.drks.de/DRKS00023911).

The project was divided into several work packages and application areas. Participants and their parents were asked to fill in a questionnaire focusing on potential behavioral strengths and difficulties of the adolescents (SDQ).

In the school and day care-centers project, COVID-19-tests were carried out at five locations (Düsseldorf, Heidelberg, Homburg, Cologne, Munich) in a total of 14 institutions. The project was implemented in 2 phases of 3 weeks each in the period from September 2020 to March 2021.

At the beginning of test phase 1 (September–December) and test phase 2 (January–March) the SDQ-questionnaire was distributed. The SDQ was used to assess psychopathological symptoms. The questionnaires were answered by the parents of children and adolescents aged 2–17 (parent ratings) and to the adolescents aged 11–17 (self-ratings). The questionnaires could be filled in as online or paper-pencil versions.

### Population

#### Study sample

The participating facilities were selected at the five participating locations. School selection aimed to cover a variety of population densities and social settings, not to be population-representative. Furthermore, the site selection was intended to reflect differences in the various German school types as well as German federal structural differences. Site recruitment required approval of communities, school boards and local health authorities.

Students needed written consent of the legal guardians. Participation was voluntary and consent could be withdrawn at any time. Adolescents aged 11–17 years who had a valid^1^ self-assessed SDQ-score as well as a valid externally assessed SDQ-score by one respective parent were included in our study population. To be considered valid, at least 80% of the (externally or self-assessed) SDQ-questionnaire needed to be completed.

No incentives were offered for participation. While *n* = 4,866 students were eligible for the B-FAST study, *n* = 1,536 had no informed consent, resulting in *n* = 3,386 students to be enrolled in the study. In total, there were *n* = 3,970 participants from 14 primary and secondary schools enrolled in the main study, including students, parents and staff.

**Figure 1:**
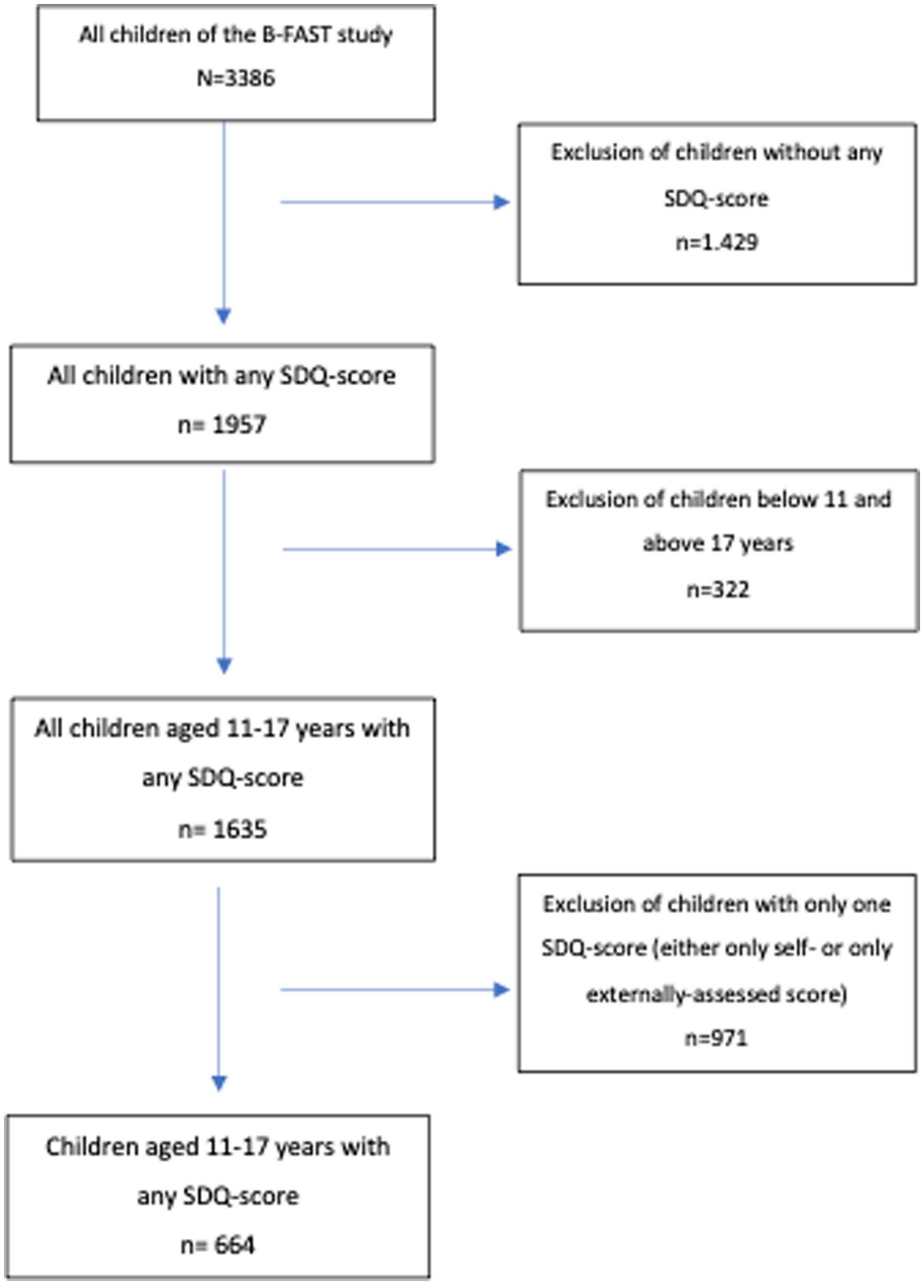
Flowchart inclusion process.

From the total study-population, *n* = 1,023 students filled in the self-assessed SDQ-questionnaire and *n* = 1,605 parents filled in the externally assessed SDQ-questionnaire. For our analyses, we only included adolescents between the age of 11 and 17 with a self-assessed SDQ-score as well as the corresponding externally assessed SDQ-score. After data cleaning, our final sample included *n* = 664 children. From these, there were *n* = 385 female, *n* = 278 male and *n* = 1 child with none specified gender. A flowchart to visualize this inclusion process can be found in Figure 1.

#### Strengths and difficulties questionnaire

The main outcome of our analysis, the behavioral strengths and difficulties of adolescents in Germany, was assessed via the SDQ. It is a validated and widely applied instrument to identify children and adolescents at risk for behavioral and mental health problems via a questionnaire that can be completed by either the child or adolescent themselves (self-assessed) or by a respective parent or teacher (externally assessed) (25). In the following, the term “externally assessed” is used in the sense of “parental assessed.”

The SDQ is composed of the following five subscales: Conduct Problems, Emotional Symptoms, Hyperactivity Symptoms, Peer Problems, and Prosocial Behavior. All subscales contain 5 questions each to be answered on a 3 step Likert scale (0 = not true; 1 = somewhat true; 2 = certainly true) (26, 27).

### Statistical analyses

Preceding the main analyses, we provide descriptive statistics of relevant covariates, namely age, gender and type of school, socioeconomic status. The type of school refers to the school types of the German school system. School education at “Hauptschule” and “Mittelschule” lasts 9 years and qualifies students for vocational training afterwards. Schooling at “Realschule” lasts 10 years and is designed for slightly academically higher-achieving students. Students at “Gymnasium” attend school for 12–13 years and graduate with the university entrance qualification (“Abitur”), which enables them to pursue further studies at universities or colleges. Admission to “Realschule” and “Gymnasium” is restricted by academic performance during elementary school. “Gemeinschaftsschulen” are a hybrid model where all students, regardless of their academic performance, learn together in common class structures. The school level refers to the year in which the respective student is currently in his/her school. Lower level consists of the years 5–6, middle level of the years 7–9 and finally high level of the years 10–12.

In a first step, each subscale was analyzed individually, with the respective five items being added together, resulting in a value range of each subscale between 0 and 10, with higher values indicating more problems. The total score (“Total Problem Score”) is calculated by adding up the values of all subscales, except the Prosocial Behavior subscale. This scale ranges from 0 to 40 and can also be interpreted independently. These scores were used for the following analyses. We compared our data to normative data from the pre-pandemic situation. These German norm values stem from the KIGGS study (28). The German KIGGS study is a cohort-sequential study that gathered comprehensive data on the health status of children and adolescents in Germany. It was part of the German health-monitoring system established at the Robert Koch Institute, Berlin, on behalf of the German Federal Ministry of Health (29). The data used for establishing German norm values by Hölling et al. for the self-assessed SDQ and for our comparison is from KiGGS wave 1, which took place between 2003 and 2006 and involved a total of 17.641 adolescents and children and their parents. Here 6.726 participants aged 11–17 years were involved. As we did not have access to the full data set, the data was simulated based on Hölling et al. (30). This enabled a comparison of our data dimensionally to the German normative data. To do so, a descriptive analysis was conducted, presenting the arithmetic means, standard deviation (SD), and the 95% confidence interval (95%CI). This was solely performed for the self-assessed data as Hölling does not provide normative data for the externally assessed values. In addition, we classified resulting scores into the categories “normal” “borderline” and “abnormal” by applying the German bandings provided by Hölling (30) to the self- assessed data as well as for the externally assessed data provided by Woerner et al. (31) and compared the results to the classification based on the United Kingdom bandings provided by Goodman (25, 32). Comparisons between male and female participants’ values of the externally assessed SDQ-scores were conducted by using the Mann–Whitney *U*-test presenting the median, the interquartile range (IQR), arithmetic mean, confidence interval and the *p*-value. Similarly, we compared external and self-assessed ratings separately for male and female participants by calculating Spearman’s correlation. Finally, as a result of the previous steps, we present the arithmetic means and 95%CI of all (sub)scales separately for participants attending Realschule or Gymnasium (school types), respectively.

The simulation of the data of the KiGGS study was performed with R, Version 4.1.2. All other analyses were performed using SPSS, Version 29.

## Results

The adolescents were on average 13.44 years old and fairly evenly distributed in terms of age groups (11–13 years: 54.8%; 14–17 years: 45.2%). Slightly more girls than boys participated (58.0%). The majority of our study participants attended “Gymnasium” (69.3%), i.e., had taken the highest possible educational path. In our sample, this was followed by “Realschule” (21.8%), which is the second highest educational path in this age group. “Hauptschule” and “Mittelschule” together accounted for only 4.4% of our cohort. “Gemeinschaftsschule” was even less represented at 0.6% (Tables 1, 2).

**Table 1:** Sample characteristics: cross-sectional population of students with both self-assessed and externally assessed (via parent) SDQ-score from November 2020 to April 2021 (*n* = 664).

Variables	*n*	%/Mean ± SD
Age in years (cont.)	664	13.44 ± 1.905
*Age in categories*		
11–13 years	365	11.95 ± 0.81
14–17 years	299	15.26 ± 1.11
*Gender*		
Male	278	41.9%
Female	385	58.0%
Not specified	1	0.2%
*Type of Secondary School Institution*		
Hauptschule (secondary modern school, graduation after year 9)	5	0.8%
Realschule (secondary school, graduation after year 10)	145	21.8%
Gymnasium (grammar school, graduation after year 12/13)	460	69.3%
Mittelschule (middle school, graduation after year 10)	24	3.6%
Gemeinschaftsschule (mixed school type)	4	0.6%
Other	25	3.8%
*School year*		
Lower grade (school year 5–6)	184	27.71%
Middle grade (school year 7–9)	316	47.59%
Higher grade (school year 10–12)	164	24.70%
Location and social structure index*		
Heidelberg	11	1.7%
Homburg	3	0.5%
Cologne	464	69.9%
Munich	186	28.0%

*n*, absolute frequencies; %, percent frequencies; SD, standard deviation.

**Table 2:** SDQ-values in simulated pre-pandemic sample versus B-fast sample during the pandemic (self-report, 11–17 years).

SDQ (sub-)scales	Report	Mean	SD	95% Confidence interval	
Emotional symptoms	Pre-pandemic*	2.43	0.09	2.38	2.48
	Pandemic	2.89	0.02	2.71	3.07
Conduct problems	Pre-pandemic	1.95	0.02	1.91	1.98
	Pandemic	1.62	0.06	1.51	1.73
Hyperactivity symptoms	Pre-pandemic	3.62	0.02	3.57	3.67
	Pandemic	3.42	0.09	3.25	3.59
Peer problems	Pre-pandemic	2.00	0.02	1.97	2.04
	Pandemic	2.12	0.64	1.99	2.24
Prosocial behavior	Pre-pandemic	7.69	0.21	7.65	7.73
	Pandemic	8.10	0.70	7.97	8.23
Total problem score	Pre-pandemic	10.02	0.07	9.86	10.16
	Pandemic	10.06	0.21	9.65	10.47

^*^Using the simulated data of the KiGGS study of the SDQ self-report based on the results by Hölling et al. (2018) with *n* = 6,726 participants, aged 11–17 years. SD, standard deviation.

**Figure 2:**
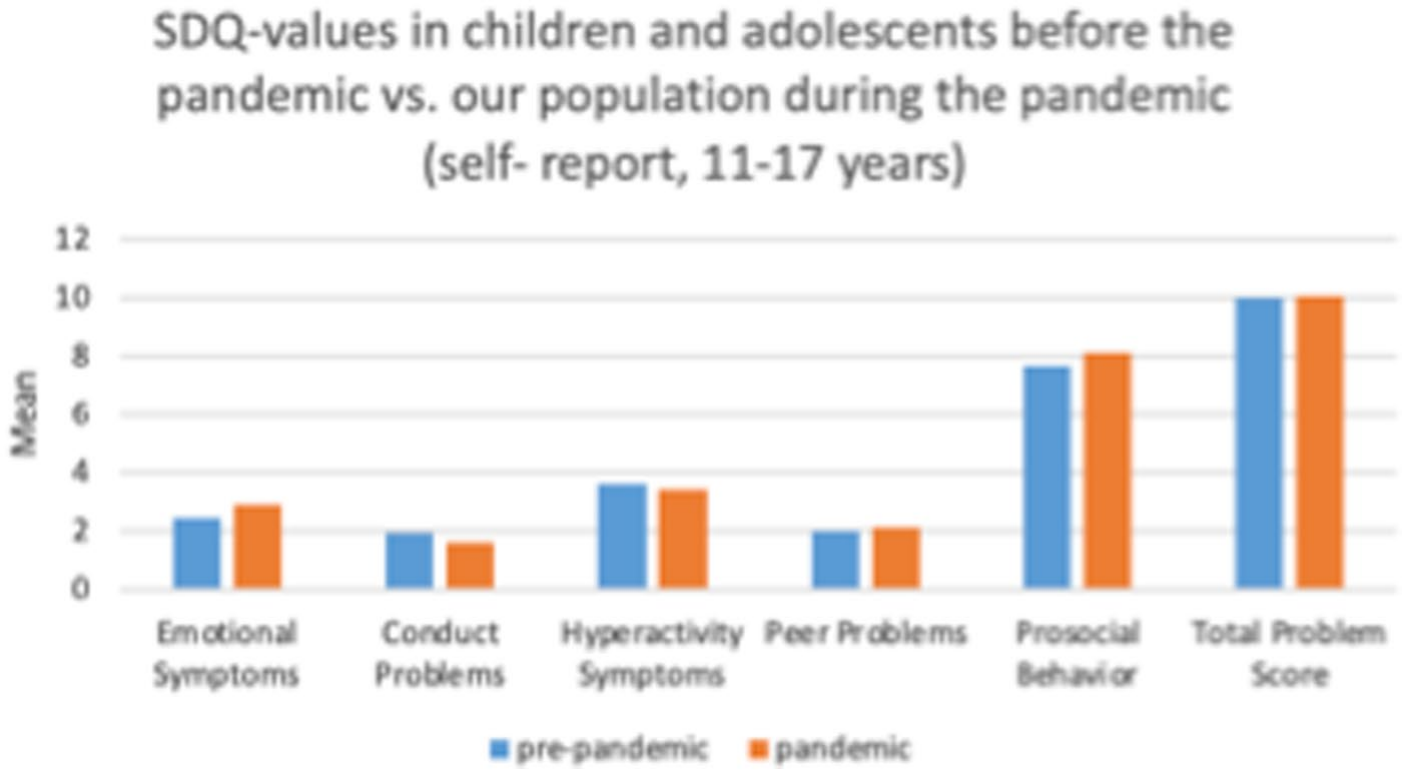
Comparison of SDQ-values.

A visualization of the comparison between SDQ-values in children and adolescents before the pandemic vs. our population during the pandemic (self-report, 11–17 years) can be found in Figure 2.

Regarding self-assessed ratings on the subscale Emotional Symptoms the pandemic-cohort is worse off than the pre-pandemic study cohort (mean = 2.89 vs. mean = 2.43). However, on the subscales Conduct Problems, Hyperactivity and Prosocial Behavior (higher values on the subscale prosocial behavior indicate a higher social competence, i.e., resources not difficulties), the pre-pandemic population rated themselves more negative than our population.

Table 3 displays the differences in results using different cut-off-values for the self-report, using German norm values based on Hölling et al. (30) and British norm values based on Goodman (25).

**Table 3:** Bandings of raw scores of self-assessed SDQ-scores using normative German data (Hölling) (30) and normative British data (Goodman) (25).

	“Normal”				“Borderline”				“Abnormal”			
	Bandings		Proportion of cases		Bandings		Proportion of cases		Bandings		Proportion of cases	
	Hölling	Goodman	Hölling	Goodman	Hölling	Goodman	Höllling	Goodman	Hölling	Goodman	Hölling	Goodman
Total Problem Score	0–14	0–15	81.60%	85.20%	15–16	16–19	6.90%	9.30%	17–40	20–40	11.50%	5.50%
Emotional Symptoms	0–4	0–5	76.70%	84.30%	5	6	7.60%	6.00%	6–10	7–10	15.70%	9.70%
Conduct Problems	0–3	0–3	90.60%	90.60%	4	4	5.10%	5.10%	5–10	5–10	4.30%	4.30%
Hyperactivity	0–5	0–5	84.50%	84.50%	6	6	6.00%	6.00%	7–10	7–10	9.50%	9.50%
Peer Problems	0–3	0–3	81.40%	81.40%	4	4–5	9.40%	14.30%	5–10	6–10	9.20%	4.30%
Prosocial Behavior	7–10	6–10	84.40%	90.20%	6	5	5.70%	5.90%	0–5	0–4	9.90%	3.90%

We found the bandings to be the same on the subscales Conduct Problems and Hyperactivity as well as on the subscale Peer Problems. On the subscales where the groupings differed, namely on the Total Problem Score, the subscale Emotional symptoms and the subscale Prosocial Behavior, we observed a larger proportion of cases labeled as “normal” when using the British bandings. Consequently, we saw a higher number of cases in the categories “borderline” and “abnormal,” when using the German bandings.

Table 4 displays the differences in results for the externally assessed report, using the German norm values based on Woerner et al. (31), and the British norm values based on Goodman (25).

**Table 4:** Bandings of raw scores of externally assessed SDQ-scores using normative German data (Woerner) (31) and normative British data (Goodman) (25).

	“Normal”				“Borderline”				“Abnormal”			
	Bandings		Proportion of cases		Bandings		Proportion of cases		Bandings		Proportion of cases	
	Woerner	Goodman	Woerner	Goodman	Woerner	Goodman	Woerner	Goodman	Woerner	Goodman	Woerner	Goodman
Total problem score	0–12	0–13	80.90%	84%	13–15	14–16	9.30%	7.20%	16–40	17–40	9.80%	8.80%
Emotional symptoms	0–3	0–3	76.20%	76.20%	4	4	10.10%	10.10%	5–10	5–10	13.70%	13.70%
Conduct problems	0–3	0–2	89.20%	77.50%	4	3	6.60%	11.60%	5–10	4–10	4.20%	10.90%
Hyperactivity	0–5	0–5	86.60%	86.60%	6	6	6.60%	6.60%	7–10	7–10	6.80%	6.80%
Peer problems	0–3	0–2	84.60%	73.30%	4	3	7.80%	11.30%	5–10	4–10	7.60%	15.40%
Prosocial behavior	6–10	6–10	89.20%	89.20%	5	5	5.40%	5.40%	0–4	0–4	5.40%	5.40%

We found the bandings to be the same on the subscales Emotional Symptoms, Hyperactivity and Prosocial Behavior. Due to different cut-offs on the Total Problem Score, we found more cases in the category “normal” and fewer cases in the categories “borderline” and “abnormal,” when using the British bandings. Also, the subscales Conduct Problems and Peer Problems use different cut-off values based on the branding, therefore we saw a larger proportion of cases in the category “normal” and a smaller proportion of cases in the category “borderline” and “abnormal” when using German bandings.

As shown in Table 5, when comparing scores between male (*n* = 278) and female participants (*n* = 385) separately for externally and self-assessed data, differences were statistically significant for the external ratings in three of the six (sub-)scales (Emotional Symptoms, Hyperactivity, Prosocial Behavior), while this was the case for four (sub)scales in the self-assessed ratings (Emotional Symptoms, Peer Problems, Prosocial Behavior, Total Problem Score). For example, on the subscale Peer Problems, sex-specific differences were statistically significant in the self-assessed but not in the external ratings (*p*-value 0.02 vs. 0.94), whereas it was the other way around for Hyperactivity symptoms (*p*-value 0.838 vs. 0.001). On the latter scale, parents rated boys more negatively than they did for girls. On the subscale Prosocial Behavior both, the externally and self-assessed scores, showed more positive ratings for girls than for boys (*p*-value parental assessed: 0.006; self-assessed: 0.001).

**Table 5:** Comparison of males and females on the self- and externally-reported SDQ-scores via Mann–Whitney *U*-test.

SDQ (sub-)scales	Report	Sex	Median	IQR	*p*-value	Mean (SE)	CI
Emotional symptoms	Parent	Male	1	0–3	<0.001	1.78 (0.11)	(1.55; 2.00)
		Female	2	1–4		2.36 (0.11)	(2.14; 2.57)
	Self	Male	2	1–5	<0.001	1.99 (0.11)	(1.77; 2.21)
		Female	3	1–5		3.55 (0.13)	(3.30; 3.80)
Conduct problems	Parent	Male	1	0–2	<0.262	1.58 (0.09)	(1.40; 1.76)
		Female	1	0–2		1.50 (0.08)	(1.34; 1.67)
	Self	Male	1	1–2	<0.390	1.56 (0.08)	(1.40; 1.73)
		Female	1	1–2		1.67 (0.08)	(1.53; 1.82)
Hyperactivity symptoms	Parent	Male	3	1–5	<0.001	3.15 (0.15)	(2.85; 3.44)
		Female	2	0–4		2.35 (0.11)	(2.13; 2.56)
	Self	Male	3	2–5	<0.838	3.42 (0.13)	(3.16; 3.67)
		Female	3	1–5		3.44 (0.12)	(3.21; 3.67)
Peer problems	Parent	Male	1	0–3	<0.943	1.61 (0.10)	(1.41; 1.81)
		Female	1	0–3		1.62 (0.09)	(1.44; 1.80)
	Self	Male	2	1–3	<0.022	1.98 (0.10)	(1.78; 2.18)
		Female	2	1–3		2.22 (0.08)	(2.06; 2.38)
Prosocial	Parent	Male	8	7–9	<0.006	7.74 (0.12)	(7.51; 7.98)
		Female	9	7–10		8.15 (0.09)	(7.96; 8.33)
	Self	Male	8	7–9	<0.001	7.70 (0.12)	(7.47; 7.93)
		Female	9	8–10		8.40 (0.08)	(8.24; 8.56)
Total problem score	Parent	Male	7	4–12	<0.407	8.11 (0.33)	(7.47; 8.75)
		Female	7	4–11		7.83 (0.29)	(7.26; 8.40)
	Self	Male	9	5–12	<0.001	8.95 (0.29)	(8.38; 9.52)
		Female	10	7–14		10.88 (0.28)	(10.32; 11.43)

The correlation coefficients between external and self-assessed ratings (Table 6) overall show a moderate to high correlation (between 0.43 for Prosocial Behavior among girls and 0.62 for peer problems among boys) with a higher consistency for boys (on average 0.55 vs. 0.49). The correlation was statistically significant in all cases.

**Table 6:** Spearman’s correlation between self- and externally assessed ratings by sex.

	Males		Females	
	Correlation coefficient	*p*-value	Correlation coefficient	*p*-value
Emotional problems	0.4386	<0.001	0.482	<0.001
Conduct problems	0.4946	<0.001	0.4628	<0.001
Hyperactivity	0.5757	<0.001	0.521	<0.001
Peer problems	0.6246	<0.001	0.5055	<0.001
Prosocial behavior	0.5815	<0.001	0.4318	<0.001
Total problem score	0.5637	<0.001	0.5414	<0.001

The school-specific presentation of arithmetic means for all scales consistently show higher values on the problem scores (see Appendix). This particularly becomes obvious in the total score 9.79 versus 6.95 (external report) and 11.67 versus 9.54 (self-report) for adolescents attending Realschule vs. Gymnasium, respectively.

## Discussion

Our main findings were (1) that in our sample, the subscale affected the most during the pandemic was the Emotional Problems subscale. In contrast, an amelioration on the subscales Conduct Problems and Prosocial Behavior was observed. We furthermore found that (2) country-specific normative data seem to be important. Our data indicated that increased emotional problems would not be detected applying other European (British) norms. Thirdly, the detailed problem profile showed sex-specific differences and differences between adolescents attending different types of school. While we found a moderate to good correlation between self-ratings and external ratings in the SDQ, our data show that both ratings should be included to obtain reliable and valid results. The SDQ is sensitive to sex and rater effects.

The present study aimed to show a dimensional problem profile of adolescents during the COVID-19 pandemic and thereby provide information on how suitable the SDQ is to measure the emotional situation of an adolescent population during a global crisis. Therefore, we used the SDQ to investigate dimensional psychopathological symptoms instead of quality of life or frequencies of categorical psychiatric disorders in a subgroup of adolescents 1 year into the SARS-CoV-2-pandemic in Germany. Both the students and their parents completed the questionnaire on psychosocial health. We observed a deterioration in our cohort on the self-assessed subscale Emotional Symptoms compared to the pre-pandemic population of the KiGGS study population (29). Similarly, comparing the classification of our sample to the classification of the pre-pandemic sample using the German cut-off-values provided by Hölling (30) for the subscale Emotional Symptoms we found a larger number of cases in the category “abnormal” (15.7% vs. 7.5%). These results are in line with a previous nationwide representative study performed during the SARS-CoV-2-pandemic showing that two thirds of the participating children and adolescents are highly burdened by the pandemic. They experienced significantly lower health related quality of life, more mental health problems, and higher anxiety levels (33). Taking into consideration that hyperactivity is more genetically determined, it is not surprising that no statistically significant changes occur on this scale before and during the pandemic. On the subscale “Conduct Problems,” we observed an improvement that is not entirely explicable based on our dataset; however, it may be attributed to alterations in daily routines (such as school closures and increased time spent at home), which potentially resulted in stress reduction for certain adolescents.

In contrast, using the bandings provided by Goodman (25), the deterioration during the SARS-CoV-2-pandemic was less accurately captured for our sample: only 9.7% are classified as “abnormal.” As a methodological consideration, the results therefore also show how different cut-offs lead to different classifications and underline the need of using country specific cut-off values (30).

Importantly, as Robert Goodman stated: “The main implication is that users probably should not be too focused on whether the score is just this side or just the other side of an arbitrary boundary. We may need to use fairly arbitrary cutoffs in terms of rules such as that above a score of X we will carry out more detailed screening, but that sort of pragmatic rule should not blind us to the fact that one point above threshold and one point below threshold actually have almost identical implications.”

Several other authors (3–11, 33) have also shown the deterioration of the mental health situation of adolescents in Germany and elsewhere. Ravens Sieberer, for example, included internationally established and validated instruments for measuring the health-related quality of life (KIDSCREEN-10), mental health problems (SDQ), anxiety (SCARED), and depression (CES-DC) (33). The deterioration of the mental health situation is not only pictured by German authors but also internationally. For example, in two Chinese studies, one in which data of Chinese primary school students were collected on depressive and anxious symptoms, non-suicidal self-injury, suicide ideation, suicide plan, and suicide attempt (34). The other, using a questionnaire, which was completed by parents, incorporating the Diagnostic and Statistical Manual of Mental Disorders (DSM-5) criteria commonly used for a cross-cultural assessment of anxiety disorders, including depression (18). Also, on a systematic review conducted by an international team from Canada, Pakistan and Australia who included 18 articles in their review with the overall finding that Children and adolescents are more likely to experience high rates of depression and anxiety during the pandemic (4).

However, on two subscales (Conduct Problems, Prosocial Behavior) our specific cohort appeared to be better off than the pre-pandemic population (30).

Bringing together our results with other studies conducted during the pandemic in Germany using the SDQ (9), our study offers further knowledge concerning mental health problems of adolescents. It does not only give insight into the externally assessed data but also self-assessed data of the adolescent. While the pre-pandemic Germany-wide BELLA study reported that 17.7% of all 7–17-year-olds are at risk for mental problems on the Total Problem Score of the SDQ (9), the COPSY study showed that this proportion of 7-17-year-olds at risk increased drastically to 30.3% (11). However, the COPSY study particularly focused on quality of life by using the KIDSCREEN and used the SDQ only to display the Total Problem Score, symptoms of depression and anxiety were generated by using different screening methods (SCARED, CES-DC, PHQ-2) (11). Our specific findings not only add knowledge regarding dimensional aspects with the full profile of the SDQ of adolescents’ well-being and psychopathology. They also give information about the consistency of ratings by parents and by the adolescents themselves. On the subscale Emotional Symptoms, for example the externally and the self-assessed SDQ-scores did not greatly deviate from each other. This confirms the existing evidence according to which externally- assessed and self-assessed SDQ-scores usually agree better as the child gets older (35, 36). The fact that we solely included adolescents, but not younger children may have contributed to this finding.

Also, equally high results for German children and adolescents were reported by the Corona Snapshot Monitoring (COSMO) study, a serial cross-sectional study designed to assess the psychosocial condition of Germans during the SARS-CoV-2-pandemic (12). Here, also approximately one-third of all under-aged children was found to be at risk for Emotional Symptoms on the SDQ scale. Still, these results were also well above pre-pandemic levels.

In contrast, the overall SDQ-scores of our cohort were more consistent with the pre-pandemic scores. For example, in the externally assessed report, merely 19% (German banding) of our cohort were classified as “at risk” on the Total Problem Score, which is rather consistent with the pre-pandemic levels of the BELLA study (9). This is also true for most externally assessed SDQ-subscale scores in our study. Except for Emotional Symptoms, 80.9–89.1% (German bandings) of all adolescents were not at risk (category “normal”), reflecting the pre-pandemic SDQ levels.

Regarding sex differences on the self- and externally assessed perceptions of adolescent mental health during the pandemic, our results are mostly consistent with previous studies (37, 38). Concerning Emotional Symptoms, girls seem to be slightly worse off than boys. For internalizing symptoms, females compared to males have been found to be more likely to react with anxiety (37). It has also been observed in a previous study that females were more likely to show symptoms of depression and anxiety than males during the SARS-CoV-2-pandemic (38). Males in our cohort were rated worse than the females solely on the externally assessed scale Hyperactivity-Symptoms. This might be due to the fact that boys are more likely to stand out with symptoms of hyperactivity (39, 40) and that these symptoms are usually more obvious and conspicuous making them more consistently discernible by parents (41, 42). On the same subscale the self-assessed scores for boys and girls demonstrate greater comparability as girls showed higher values in the self-ratings compared to their parents’ ratings. There is a lot of evidence supporting higher hyperactivity among boys than among girls (39), leading to the conclusion that self-ratings for hyperactivity might not be reliable.

However, a similar picture with self-ratings resulting in higher values than external ratings particularly in females, could be observed for emotional symptoms (2.36 vs. 3.55) and peer problems (1.62 vs. 2.22). This pattern is in line with previous literature, which indicates that self-ratings tend to reflect more problematic perceptions compared to external ratings (25, 30, 43), highlighting the importance of surveying adolescents themselves, as parents may not be aware of everything. Consequently, this not only makes our data plausible and is a confirmation of previous findings from pre-pandemic times. Furthermore, according to our data, this pattern seems to have persisted during the pandemic, even though adolescents spent significantly more time at home during this period.

In attempting to elucidate the observed sex differences, it is imperative to consider pre-pandemic research findings, emphasizing that these disparities are not solely pandemic-specific. These antecedent studies generally corroborate our observations regarding sex disparities amidst the pandemic. Numerous investigations support the differential manifestation of ADHD and anxiety disorders in boys and girls. Biologically, variations in neurobiological functioning and hormonal regulation are posited as potential contributing factors. Moreover, gender-specific social dynamics and patterns of upbringing are proposed to exert influence over the prevalence of these psychiatric conditions (44–47).

We found the SDQ to be a reliable tool to measure strengths and difficulties of adolescents during a crisis such as a global pandemic. From our data, it seems that adolescent-centered interventions should focus on emotional and psychosomatic but also externalizing difficulties and should consider that males and females might react differently to stressful situations and show different symptoms. In the light of the children and adolescents-focused mental health surveillance program planned by the central German public health institute, the Robert Koch Institute (RKI) (48), these findings could contribute to a potential starting-point to draw meaningful conclusions for a mental health monitoring strategy in future crisis situations. Also, we found no existing normative German data to sufficiently compare parents and their children using the SDQ. We merely found normative German data for the self-assessed SDQ and in another study conducted on another population normative German data for the externally assessed SDQ. Therefore, our findings help to show that there are corresponding normative German data on the self- and the externally assessed SDQ missing. We recommend conducting a representative study to generate normative data for both the externally and self-assessed SDQ-scores.

In addition, our study also provides insight into who can be reached with surveillance studies in schools during a time of crisis. Vulnerable groups seem to be insufficiently addressed by these participation offers and thus are hardly included, which leads to a major bias conflict of such study endeavors. In this respect, our experiences should provide an impulse to develop strategies for data collection at schools in order to prevent this problem in the future and to be able to represent the German general population more reliably.

### Strengths and limitations

This study, despite its well-designed multicenter efforts of study facilities, universities and research institutes, has some limitations. Thus, it is likely that a selection bias of the participants led to a systematic bias of the study cohort, where the considered adolescents seem to be more educated, urban and financially better off than the German average. The SDQ-Questionnaire is available in over 80 languages; however, in this study it was solely administered in German. Consequently, the cohort is likely to have a lower proportion of participants with migration background compared to the general population. In addition, the uneven geographic distribution of study participants was influenced by the distribution of study centers. For example, no data collection took place at schools in northern or eastern Germany. Other potential homogeneities within our study sample, such as specific participation motivation or beliefs, could not be accounted for at all.

Therefore, the external validity of our results is restricted and can only be transferred to the general population in a limited way. Furthermore, as a cross-sectional approach, this study represents only a snapshot of the mental situation of adolescents during the SARS-CoV-2-pandemic. Furthermore, the cross-sectional design is a limitation of this study. It stems from the primary focus of the overarching B-Fast study, which was primarily concerned with testing strategies within schools. However, our paper effectively leveraged secondary outcomes from the main study to present a valuable snapshot of the mental well-being of adolescents during the SARS-CoV-2-pandemic.

In our sample social distancing seems to lead to reduced conduct problems or hyperactive behavior at school. This might be in part due to a selection bias in our cohort. The specific distribution of students across school institutions suggests that there is some middle-class bias in our study population. Good parental support and sufficiently large houses with gardens mitigate short-term negative effects of social distancing. In the 2018/2019 Germany-wide quota for the different school types, students of “Gymnasium” make up the largest share on average across Germany, but they only do so between 35% (49) and around 50% including “Fachhochschulreife” (=higher education entrance qualification for universities of applied sciences) (50), which is considerably less than in our study cohort. Certain social disparities are known to exist with regard to school transfer to a secondary school, so that children from financially stronger, more educationally advantaged families have a greater chance of attending “Gymnasium,” i.e., the highest possible educational path, than children from working-class families, even with the same aptitude (51). It can therefore be assumed that our cohort belongs to middle class at an above-average rate and is better off financially, socially and educationally than the average German population.

In terms of geographic distribution, it is striking that 69.9% of the participants were based in Cologne, 28.1% in Munich. Thus, our cohort is also more urban and metropolitan than the German average.

Evidence shows that socioeconomic factors such as lower income (52), migration status (9), or single parent status (53–56) are correlated with higher stressors and correspondingly poorer general mental health during the pandemic. These vulnerable groups were probably very underrepresented in our sample, which may have influenced our results. Therefore, our specific findings concerning Conduct Problems and Prosocial Behavior and those concerning scales with no changes might partly be related to the selection bias comprising the external validity of the study. More specifically, our cohort was less affected by the pandemic situation or it was better protected by resources and protective factors (e.g., financial stability and social support system). However, the deterioration on the subscale Emotional Symptoms is unlikely to be explained by the selection bias. It remains open whether in a representative sample, the deterioration on this subscale would be even stronger or whether this subscale mirrors in particular the problems of adolescents with a higher SES.

## Conclusion

This study offers a noteworthy glimpse into the strengths and challenges experienced by adolescents in Germany during the first year of the SARS-CoV-2-pandemic. By delineating distinct problem profiles for boys and girls, we observed a remarkable impact on emotional symptoms, while hyperactivity, largely determined by genetic factors, showed less susceptibility to the pandemic’s effects. In addition, as methodological implications, the study underscores the importance of utilizing both self-reported and external assessments and emphasizes the necessity of employing country-specific cut-off values. Moreover and importantly, it reaffirms the utility of the SDQ as a valuable assessment tool, even within the unique circumstances posed by a pandemic.

Health and practical implications:

- The effort to collect both, external and self-assessed questionnaires, is worthwhile.
- The SDQ provides a comprehensive overview of psychiatric symptoms and effectively captures their changes within the context of the SARS-CoV2-pandemic. Therefore, the SDQ is a valid tool for assessing psychiatric symptoms during a pandemic.
- In our sample we saw a deterioration on the subscale emotional symptoms. The focus of a potential therapy could be directed towards addressing these symptoms. This might include approaches such as psychotherapy, cognitive-behavioral techniques and mindfulness-based interventions, emotion regulation training, and family therapy which have shown promise in targeting these needs. Further, using more specific questionnaires can enhance the assessment of symptoms, enabling clinicians to tailor interventions more effectively and to improve research. Gender specific differences should be considered in psychiatric treatment.

## Data availability statement

The original contributions presented in the study are included in the article/Supplementary material, further inquiries can be directed to the corresponding author: stephan.bender@uk-koeln.de.

## Author contributions

JL: Writing – original draft, Writing – review & editing, Conceptualization, Formal analysis, Methodology, Supervision. JK: Writing – original draft, Writing – review & editing, Methodology. JD: Writing – review & editing. JF: Writing – review & editing, Data curation, Formal analysis, Methodology. SB: Writing – review & editing, Supervision, Conceptualization, Funding acquisition, Writing – original draft.
