# Supplementary material for: Exploring adolescent mental health during the COVID-19 crisis – strengths and difficulties
Source: Front Public Health. 2024 Apr 4;12:1357766. doi: 10.3389/fpubh.2024.1357766 (PMC11024382; doi:10.3389/fpubh.2024.1357766)
Supplement: Supplementary file 1 [file Table_1.DOCX]

**SDQ values of adolescents (11-17y) of externally and self- assessed scores in Realschule and Gymnasium**

| SDQ (Sub)-scales | Type of school | Mean | SD | 95 % Confidence Interval | |
| --- | --- | --- | --- | --- | --- |
| Emotional Symptoms (self report) | Realschule | 3.13 | 0.22 | 2.71 | 3.56 |
|  |  |  |  |  |  |
|  | Gymnasium | 2.88 | 0.11 | 2.66 | 3.10 |
|  |  |  |  |  |  |
| Conduct Problems (self report) | Realschule | 1.94 | 0.14 | 1.67 | 2.21 |
|  |  |  |  |  |  |
|  | Gymnasium | 1.51 | 0.06 | 1.39 | 1.63 |
|  |  |  |  |  |  |
| Hyperactivity Symptoms (self report) | Realschule | 3.45 | 0.16 | 3.12 | 3.77 |
|  |  |  |  |  |  |
|  | Gymnasium | 3.35 | 0.11 | 3.14 | 3.56 |
|  |  |  |  |  |  |
| Peer Problems (self report) | Realschule | 3.15 | 0.15 | 2.85 | 3.45 |
|  |  |  |  |  |  |
|  | Gymnasium | 1.80 | 0.07 | 1.66 | 1.93 |
|  |  |  |  |  |  |
| Prosocial Behavior (self report) | Realschule | 7.63 | 0.17 | 7.29 | 7.98 |
|  |  |  |  |  |  |
|  | Gymnasium | 8.28 | 0.07 | 8.13 | 8.43 |
|  |  |  |  |  |  |
| Total Problem Score (self report) | Realschule | 11.67 | 0.48 | 10.73 | 12.61 |
|  |  |  |  |  |  |
|  | Gymnasium | 9.54 | 0.24 | 9.07 | 10.01 |
|  |  |  |  |  |  |
| Emotional Symptoms (parent report) | Realschule | 2.29 | 0.19 | 1.91 | 2.67 |
|  |  |  |  |  |  |
|  | Gymnasium | 2.00 | 0.09 | 1.81 | 2.18 |
|  |  |  |  |  |  |
| Conduct Problems (parent report) | Realschule | 1.83 | 0.16 | 1.53 | 2.14 |
|  |  |  |  |  |  |
|  | Gymnasium | 1.39 | 0.07 | 1.26 | 1.52 |
|  |  |  |  |  |  |
| Hyperactivity Symptoms (parent report) | Realschule | 3.06 | 0.18 | 2.70 | 3.42 |
|  |  |  |  |  |  |
|  | Gymnasium | 2.37 | 0.10 | 2.17 | 2.90 |
|  |  |  |  |  |  |
| Peer Problems (parent report) | Realschule | 2.61 | 0.15 | 2.32 | 2.90 |
|  |  |  |  |  |  |
|  | Gymnasium | 1.19 | 0.07 | 1.05 | 1.32 |
|  |  |  |  |  |  |
| Prosocial Behavior (parent report) | Realschule | 7.70 | 0.17 | 7.37 | 8.03 |
|  |  |  |  |  |  |
|  | Gymnasium | 8.12 | 0.09 | 7.94 | 8.29 |
|  |  |  |  |  |  |
| Total Problem Score (parent report) | Realschule | 9.79 | 0.52 | 8.77 | 10.81 |
|  |  |  |  |  |  |
|  | Gymnasium | 6.95 | 0.23 | 6.49 | 7.40 |
|  |  |  |  |  |  |

*Note: SD= Standard deviation*
